# Investigating the factor structure of the Montreal Cognitive Assessment: a qualitative review

**DOI:** 10.3389/fpsyg.2025.1727202

**Published:** 2025-12-17

**Authors:** Maria Rita Sergi, Michela Balsamo, Giorgia D’Ignazio, Michela Terrei, Rocco Palumbo, Gianpaolo Salvatore, Leonardo Carlucci

**Affiliations:** 1 Department of Psychology, University of G.d’Annunzio, Chieti-Pescara, Chieti, Italy; 2 Department of Psychology and Health Sciences, Pegaso Telematic University, Naples, Italy; 3 Department of Humanities, Letters, Cultural Heritage and Educational Studies, University of Foggia, Foggia, Italy

**Keywords:** Montreal Cognitive Assessment (MoCA), factor structure, Item Response Theory (IRT), MCI, dementia

## Abstract

**Introduction:**

The Montreal Cognitive Assessment (MoCA) is one of the most widely used screening instruments for Mild Cognitive Impairment (MCI) and dementia. Despite its popularity, uncertainty remains regarding its factorial structure and psychometric functioning across populations and cultures. This review aims to critically evaluate the factorial validity and dimensionality of the MoCA through Classical Test Theory (CTT) and Item Response Theory (IRT) models.

**Method:**

Following the PICO framework, a qualitative review was conducted using PubMed, Web of Science, PsycINFO, and Google Scholar. Inclusion criteria consisted of peer-reviewed empirical studies employing exploratory or confirmatory factor analyses, as well as IRT in samples of older adults.

**Results:**

Across CTT studies, findings ranged from two-factor to hierarchical multi-factor models, with a general cognitive factor frequently emerging. IRT analyses generally supported a unidimensional latent structure, identifying Executive Function, Visuospatial, and Language items as the most discriminative, while Orientation and Memory showed low discriminative power.

**Conclusion:**

Our results showed that the MoCA primarily measures a general cognitive dimension, reflecting variable contributions from different cognitive domains. Standardizing scoring metrics and ensuring cross-cultural factorial equivalence are essential to enhance the tool’s accuracy and interpretation of its score.

## Introduction

Dementia is a global health and economic challenge, costing USD 1.31 trillion in 2019, with half of this from family care, and expected to exceed USD 2.8 trillion by 2030 (World Health Organization, 2025). In Italy, the annual economic burden amounts to around € 23 billion, 63% of which is borne directly by families. Given these substantial costs, implementing efficient cognitive screening programmes is crucial for enabling the early detection of cognitive decline (Italian National Institute of Health, 2025). A timely diagnosis can facilitate interventions that delay progression, reduce the need for institutionalization, and improve quality of life for patients and their caregivers (Cova et al., 2022; Terracciano et al., 2014, 2017).

The term “*stable MCI”* has been introduced to describe forms of MCI that do not exhibit significant impairment in daily functioning. Evidence suggests that the preservation of everyday activities is associated with several protective factors, including higher educational attainment, regular engagement in intellectual and physical activities, greater brain reserve, and the absence of major depressive or other mood disorders. These factors are frequently linked to non-progression to dementia (Lee, 2023; Richard and Brayne, 2014).

Consequently, early identification of MCI, the timely implementation of targeted intervention strategies, and accurate assessment of cognitive functioning are essential for delaying or preventing the progression to dementia (Ciesielska et al., 2016).

Mild Cognitive Impairment (MCI) has traditionally been viewed as an intermediate stage between normal cognitive aging and dementia, characterized by cognitive decline that is not insufficiently severe to compromise independence in daily activities (Petersen et al., 1999). The construct of MCI serves as a diagnostic umbrella encompassing multiple perspectives, including both the preclinical dementia framework and a more strictly nosological or conceptual definition (Bermejo-Pareja et al., 2021; Lee, 2023).

Within the preclinical dementia perspective, MCI is characterised by a significant cognitive decline from a previous level of performance in one or more cognitive domains (attention, executive function, learning and memory, language, perceptual-motor, or social cognition) (American Psychiatric Association, 2022). Although MCI has often been described as a potential precursor to dementia, this association is not uniformly supported across studies (Öksüz et al., 2024; Salemme et al., 2025; Thaipisuttikul et al., 2022). When considered within a purely nosological framework, in fact, MCI does not necessarily entail a progressive trajectory toward dementia.

The Mini-Mental State Examination (MMSE) (Folstein et al., 1975) and the Montreal Cognitive Assessment (MoCA) (Nasreddine et al., 2005) are among the most widely used screening instruments for the detection of MCI and dementia in both clinical and research settings (Arevalo-Rodriguez et al., 2015; Fernandes et al., 2021; Limongi et al., 2019). Several additional tools are available for cognitive screening, including the Addenbrooke’s Cognitive Examination Revised (ACE-R) (Mioshi et al., 2006), the Mini-Cog Test (Borson et al., 2000), and the Informant Questionnaire on Cognitive Decline in the Elderly (IQCODE) (Jorm and Jacomb, 1989). However, the psychometric properties of many of these instruments have received relatively limited attention in the scientific literature (Tsai et al., 2012).

Within this context, the literature has highlighted two major limitations of the MMSE: insufficient sensitivity—particularly in detecting early cognitive decline—and weak correlations between total scores and demographic variables such as age, education, and gender (Karimi et al., 2022). These limitations have stimulated the development of alternative assessment instruments, such as the Repeatable Battery for the Assessment of Neuropsychological Status (RBANS) (Randolph et al., 1998) and The Consortium to Establish a Registry for Alzheimer’s Disease (CERAD) (Morris et al., 1989).

The MoCA is a 30-point screening measure that assesses multiple cognitive domains, including Visuospatial abilities (Trial Making Test, Cube, Clock-Drawing task; score range 0–4), Attention (Forward and Backward digit span; score range 0–6), Language (Naming, Repetition, and Phonemic Fluency), Memory (Recall; score range 0–5), Abstraction (Means of similarities; score range 0–2), and Orientation (Date, Month, Year, Place, and City; score range 0–6).

The ease of administration, brief administration time, and superior diagnostic accuracy compared to traditional instruments such as the MMSE have contributed to the MoCA’s widespread use in both clinical and research contexts (Dautzenberg et al., 2020; Jia et al., 2021; Malek-Ahmadi and Nikkhahmanesh, 2024). Beyond its psychometric strengths, the MoCA is also widely recognised for its strong clinical applicability. It can be easily administered with minimal burden by trained clinicians or healthcare professionals across a variety of heathcare settings, supporting the early screening of cognitive impairment and assisting differential diagnosis (Dautzenberg et al., 2020; Julayanont et al., 2013).

Despite its advantages, the MoCA also presents several psychometric limitations. For instance, Cova et al. (2022) identified 86 culturally adapted versions of the test, highlighting substantial variability in its cross-cultural implementation. In major English-speaking countries (US, UK, Australia), the original Canadian version is predominantly used, often without cross-cultural adaptations. In Latin America, two Spanish-language adaptations (Chilean and Argentine) are commonly employed.

In contrast, only a limited number of validated versions are available in the African context, whereas Asia has produced the largest number of culturally adapted versions.

As noted by Santangelo et al. (2015), the use of multiple MoCA cut-off scores presents several limitations, including an increased risk of misclassification—whereby the same score may be interpreted differently depending on the applied context— reduced comparability across studies, and variability in diagnostic accuracy.

Furthermore, translation alone does not ensure psychometric equivalence; culturally adapted versions may differ in diagnostic accuracy, sensitivity, specificity, and reliability.

International research has examined the the diagnostic accuracy of the MoCA in both clinical and non-clinical samples, highlighting substantial variability in recommended cut-off scores (Cova et al., 2022). Within the Italian population, several distinct cut-off points have been proposed, including 14 (Bosco et al., 2017), 26 (Conti et al., 2015), 22 (Fiorenzato et al., 2024), 15 (Pirrotta et al., 2015; Santangelo et al., 2015). At the international level, reported cut-off values show similarly wide variability, ranging from 17 to 25 across different studies and populations (Freitas et al., 2013; Hernández-Medrano et al., 2025; Karimi et al., 2022; Krishnan et al., 2017; Sun et al., 2023; Tan et al., 2014; Wang et al., 2019).

Validation studies have often assessed the diagnostic accuracy of the MoCA without investigating its factorial validity (Pirrotta et al., 2015). Pirani et al. (2022) found optimal reliability and a good diagnostic accuracy in discriminating normal cognition from early impairment; however, they did not provide information regarding item translation nor did they investigate the instrument’s factor structure.

Evidence on MoCA dimensionality remains mixed: while several studies have supported a unidimensional model (Aiello et al., 2022; Arcuri et al., 2015; Brandão et al., 2025; D’Iorio et al., 2023; Freitas et al., 2014), others have proposed multifactorial structures (Benge et al., 2017; Coen et al., 2016; Duro et al., 2010; Freitas et al., 2012; Sala et al., 2020; Smith et al., 2020).

Adaptation procedures must align with the operational definition of the construct being measured (Howieson, 2019; Nguyen et al., 2024). Cognitive assessments should follow internationally recognized guidelines for test adaptation, which ensure semantic, conceptual, and metric equivalence between the original instrument and its adapted version (Hemrungrojn et al., 2024; Waheed et al., 2020). Consequently, effective translation and adaptation require the involvement of professionals who are familiar both with the culture of origin and with the psychological constructs assessed by the instrument.

Adaptation procedures must align with the operational definition of the construct being measured (Howieson, 2019; Nguyen et al., 2024). Cognitive tests should follow internationally recognized adaptation guidelines that guarantee semantic, conceptual, and metric equivalence between the original instrument and its adapted version (Hemrungrojn et al., 2024; Waheed et al., 2020). Therefore, achieving effective translation and adaptation requires the involvement of professionals familiar with both the source culture and the constructs assessed by the test.

A pioneering study on the guidelines for the adaptation and translation of the MoCA (Khan et al., 2022) identified seven key phases: translation of the instrument into the target language without cultural adaptation, and back-translation, collection of participant feedback on item comprehension; expert recommendations, expert review; item refinement based on recommendations; consultation with the original authors; and pilot study. The authors reported that language and memory items were those most extensively culturally adapted, whereas attention and visuospatial items remained largely unchanged. In the Italian context, however, only the first step of these guidelines has been implemented in the available MoCA adaptation (Pirrotta et al., 2015), which focused solely on diagnostic accuracy.

The heterogeneity of findings across studies may be attributed ed. to multiple methodological factors, including differences in samples characteristics (clinical condition, age, and educational level), versions of the MoCA employed, and statistical criteria used to examine the factor structure. Such variability is not be unique to the MoCA; many neuropsychological tools present similar challenges (Howieson, 2019). Indeed, these tools have been criticized for several methodological and psychometric limitations (Nguyen et al., 2024). Many were initially developed in clinical and experimental settings to meet specific assessment needs and were only subsequently subjected to rigorous psychometric evaluation (Eling, 2019). As a result, they frequently fail to meet the International Test Commission Guidelines (2017), particularly in terms of standardization and construct validity, which can contribute to discrepancies in findings across studies and cultural contexts.

The coexistence of multiple normative data within the same national context could further complicates the identification of true positives and false negatives. Moreover, many commonly used neuropsychological tests lack normative data in several countries (e.g., France, Greece, Ecuador, and Canada), leading clinicians to rely on normative data developed elsewhere (delCacho-Tena et al., 2024).

For example, more than 50% of clinicians in France, Canada, and Ecuador, and over 30% of clinicians in Greece reported using normative data from other countries due to this limitation. Another major challenge of neuropsychological instruments concerns construct equivalence. Indeed, translation alone is not sufficient to ensure the validity of a tool. Only in-depth psychometric testing can confirm whether the underlying construct is equivalent across different languages and cultures (delCacho-Tena et al., 2024; Howieson, 2019; Nguyen et al., 2024).

Understanding the MoCA’s psychometric properties is thus essential to refining assessment practices, improving differential diagnosis, and enhancing the tool’s cultural and linguistic adaptability.

In particular, understanding the structure of the MoCA also serves to verify construct validity, confirm the correspondence between theory and measurement, reduce redundancy or semantic overlap (Benge et al., 2017), and support the development of reliable instruments. It also provides the foundation for the application of advanced statistical models, such as confirmatory factor analysis, (CFA), Item Response Theory (IRT), and Structural Equation Modeling (SEM) (Cronbach and Meehl, 1955; DeVellis and Thorpe, 2022; Fabrigar et al., 1999; Floyd and Widaman, 1995; Goretzko et al., 2021; Kline, 2015; Li and Savalei, 2025; Stefana et al., 2025). A well-defined factor structure demonstrates that items accurately reflect the theoretically predicted latent domains because without such evidence, score interpretation can be misleading (Sellbom and Tellegen, 2019). Additionally, testing measurement invariance is crucial for determining whether the tool’s structure remains stable across different populations (El-Den et al., 2020; Fenn et al., 2020).

Therefore, it is fundamental for researchers and clinicians to ensure that an adapted version of a test maintains content, criteria, and construct equivalence with respect to the original instrument. Inadequate adaptation procedures can introduce substantial sources of biases, ultimately compromising both diagnostic accuracy and validity of the instrument (Whittington et al., 2022). Deriving a unidimensional total score from a multidimensional measurement instrument is methodologically problematic, because it aggregates conceptually distinct cognitive domains, thereby violating the assumption of construct homogeneity (Balsamo and Saggino, 2014; Balsamo et al., 2014).

When this occurs, studies examining sensitivity and specificity analyses based on a single total score may yield attenuated or misleading estimates of diagnostic accuracy (Garrido et al., 2019; Whittington et al., 2022). This issue is particularly relevant for the MoCA, whose total score is calculated as the sum of all items despite persistent uncertainty regarding whether its underlying structure is unidimensional or multidimensional. According to Kline (2005), a single total score is psychometrically valid only when the items contributing to it share a common metric, structure, and dimensionality. Assessing dimensionality may require advanced item-level methods such as IRT (DeVellis and Thorpe, 2022; Embretson and Reise, 2013; Li, 2016; Preston and Colman, 2000; Raykov and Marcoulides, 2011; Schmucker and Moore, 2025; Thorpe and Favia, 2012; Wallmark et al., 2024). IRT is characterized by the interaction between the latent trait and psychometric functioning of individual items, while methods based on the Classical Test Theory (CTT) analyzed measurement properties primarily at the test level. Unlike CFA or CTT-based approaches, IRT provides item-level precision by estimating parameters such as item difficulty and discrimination, and by modelling measurement error as a function of the latent trait assuming it is constant. Among IRT models for polytomous items, the Graded Response Model (GRM) (Samejima, 2016) and the Partial Credit Model (PCM) (Masters, 2016) are among the most widely applied (Embretson and Reise, 2013; Stefana et al., 2025).

The cultural adaptation of a test enables the collection of local normative data, the evaluation of its factor structure and reliability, and the verification of the stability of this structure within the target population (Hambleton et al., 2004; Van de Vijver and Tanzer, 2004). The cultural adaptation of the testing allows local normative data to be collected, the factor structure and the reliability to be analysed, and ensure that the factor structure is stable in the target population (Hambleton et al., 2004; Van de Vijver and Tanzer, 2004). To achieve these goals, well-established guidelines should be followed, including standardized translation procedures, norm adjustment, and local psychometric validation studies (Waheed et al., 2020). To date, no systematic review has examined the factorial validity of the MoCA exists. Addressing this critical gap would help clarify the instrument’s internal structure and enhance its clinical interpretability. Therefore, the aim of the present review is to critically analyze the evidence on the MoCA’s factor structure across both clinical and non-clinical samples.

## Method

Our research question was formulated using the PICO model: Population (target samples assessed with MocA), Intervention (psychometric evaluations including CTT and IRT analyses), Comparison (alternative factor structures, estimation methods, or model specifications), and Outcome (fit indeces and item level parameters) (Methley et al., 2014). The qualitative review was conducted using the most important scientific databases, including Google Scholar, PubMed, Web of Science, and PsycINFO.

The search strategy was based on the following terms: “MOCA” [Title/Abstract] OR “Montreal Cognitive Assessment” [Title/Abstract] OR “short form” [Title/Abstract] OR “s-MoCA” [Title/Abstract]) AND (“factor structure” [Title/Abstract] OR “factor analysis” [Title/Abstract] OR “psychometric properties” [Title/Abstract] OR “construct validity” [Title/Abstract] OR “validation” [Title/Abstract].

The inclusion criteria were: 1. Empirical, peer-reviewed studies on the MoCA published in English; 2. Studies employing one or more the following approaches to evaluate psychometric properties: (a) Exploratory Factor Analysis (EFA); (b) Confirmatory Factor Analysis (CFA); (c) Item Response Theory (IRT); 3. Studies involving older adults aged 65 years or older.

The exclusion criteria were: 1. studies on the MoCA lacking empirical data; 2. studies involving pediatric and younger adult populations.

## Results

### Classical test theory (CTT) results

Table 1 summarizes the factor models of the MoCA based on CTT. An early study by Duro et al. (2010) proposed a two-factor model that differentiates between Memory/Language/Orientation and Attention/Visuospatial/ Executive domains. Their results showed good fit indices, Chi-square χ^2^_(7)_ = 13.996, *p* = 0.05; Comparative Fit Index (CFI) = 0.985; Root Mean Square Error of Approximation (RMSEA) = 0.069 (Hu and Bentler, 1999).

**Table 1:** Factor structure of the MoCA using classical test theory.

Study	P-population	I-intervention	C-comparison	O-outcome
Duro et al. (2010)	Total sample Age:71.78 ± 9.11 *N* = 212 *N* = 82 MCI *N* = 70 AD *N* = 25 VaD *N* = 35 ODD	CFA	Two correlated factor model: (1) Memory which included Memory, Language and Orientation domains; (2) Attention/Executive Functions.	CFI = 0.985, RMSEA = 0.069
Freitas et al. (2012)	Total sample *N* = 212 Age:59.4 ± 15.5 *N* = 90 MCI *N* = 90 AD	CFA WLS estimator	One-factor second order model: (1) Executive Functions, (2) Language, (3) Visuospatial Skills, (4) Short-term Memory, (5) Attention/Concentration/Working Memory, and (6) Orientation. Higher-order global cognition factor named “Cognition.”	CFI = 0.969, RMSEA = 0.033
Coen et al. (2016)	*N* = 2.342 community-dwelling adults Age:72.64 ± 6.19	CFA WLS estimator	One-factor second order model: (1) Executive Functions, (2) Language, (3) Visuospatial Skills, (4) Short-term Memory, (5) Attention/Concentration/Working Memory, and (6) Orientation. Higher-order global cognition factor named “Cognition.”	CFI = 0.990, RMSEA = 0.013
Sala et al. (2020)	N = 2.408 older adults organized into three cohorts: 69–71 Age: 24.03 ± 3.66 79–81 Age: 22.28 ± 4.29 89–91 Age: 20.24 ± 5.15	CFA WLS estimator.	Hierarchical factorial structure with a general factor + 7 lower-order factor: (1) Executive Functions, (2) Language, (3) Visuospatial Skills, (4) Short-term Memory, (5) Attention/Concentration/Working Memory, (6) Orientation, and (7) Abstraction. Higher-order global cognition factor named “Cognition.”	CFI = 0.970, RMSEA = 0.019
Benge et al. (2017)	*N* = 357 PD Age: 67.6 ± 9.2	EFA	Three correlated factors: (1) Attention/Executive Functions, (2) Memory, (3) Verbal Attention	
Smith et al. (2020)	*N* = 1738 PD Age: 67.6 ± 9.2	CFA ML estimator	Three correlated factors: (1) Executive Functions, (2) Memory, (3) Verbal Attention	CFI = 0.810, RMSEA = 0.093
D’Iorio et al. (2023)	*N* = 86 with focal dystonia Age: 60.1 ± 11	PCA	Mono-component structure accounting for 38.41% of variance	
Karim and Venkatachalam (2022)	*N* = 104 HG *N* = 129 MCI Age: ≥ 60	CFA	Six correlated factors: (1) Executive Functions, (2) Language, (3) Visuospatial Skills, (4) Short- term Memory, (5) Attention/Concentration, and (6) Orientation	CFI = 0.950, RMSEA = 0.058

RMSEA, root mean square error of approximation; CFI, Comparative Fit Index; HG, Healthy Group; AD, Alzheimer’s Disease; PD, Parkinson’s Disease; MCI, Mild Cognitive Impairment; FTD, Frontotemporal dementia; VaD, Vascular dementia; ODD, Other Degenerative Dementia; CFA, Confirmatory Factor Analysis; EFA, Exploratory Factor Analysis; PCA, Principal Component Analysis; CI, Confidence Interval; WLS, Weighted least square estimation; ML, Maximum Likelihood.

Other studies have supported a second-order model in which the six first-order cognitive domains loaded onto a higher-order global cognition factor (Coen et al., 2016; Freitas et al., 2012; Karim and Venkatachalam, 2022). The six first-order cognitive factors correspond to those originally proposed by Nasreddine et al. (2005): Executive Functions, Language, Visuospatial Skills, Short-term Memory, Attention/Concentration/Working Memory, and Orientation.

Further support for a hierarchical structure was provided by Sala et al. (2020), who identified seven cognitive domains loading onto a general cognitive factor in a large community-dwelling elderly sample. The study also tested the measurement invariance across demographic variables. The cognitive factors included Executive Function, Visuospatial abilities, Attention/Concentration, Language, Abstraction, Short-term Memory, and Orientation. Model fit indices indicated a good fit: χ^2^_(317)_ = 453.776 (*p* < 0.01), CFI = 0.970, RMSEA = 0.019. Other studies have reported inconsistent findings. Specifically, Benge et al. (2017) identified a three-factor solution (Executive Dysfunction, Memory, Verbal Attention) in patients with Parkinson’s disease. The factor model was tested using EFA with Promax rotation. In contrast, Smith et al. (2020) tested several factorial structures and found that the three-factor model (Executive Dysfunction, Memory, Verbal Attention) showed the best fit, with χ^2^_(101)_ = 1623.26, (*p* < 0.001), CFI = 0.81, and RMSEA = 0.093. A six-factor model, including Short-term Memory, Visuo-Spatial Abilities, Attention/Working Memory, Executive Function, Language, and Orientation, was also identified. This model showed the following fit indices: χ^2^_(155)_ = 2753.84, (*p* < 0.001), CFI = 0.74 and RMSEA = 0.098. A second-order model was also identified, which the six first-order cognitive domains loaded onto a global cognitive factor, yielding χ^2^_(164)_ = 2994.20, *p* < 0.001, CFI = 0.72, and RMSEA = 0.100. In the Italian context, D’Iorio et al. (2023) examined the MoCA’s unidimensional structure using Principal Component Analysis (PCA) in a sample of 86 adult patients with focal dystonia. In their study, the total score was employed as a diagnostic indicator.

### Item Response Theory (IRT) results

Table 2 showed the factor structure of the MoCA through IRT. Most studies reported an unidimensional latent structure (Aiello et al., 2022; Arcuri et al., 2015; de Paula Brandão et al., 2025; Freitas et al., 2014).

**Table 2:** Item-level psychometric properties of the MoCA using Item Response Theory.

Study	P-population	I-intervention	C-comparison	O-outcome
Freitas et al. (2014)	*N* = 897 Total sample Age: 60.3 ± 15.4 *N* = 650 HG Age: 55.8 ± 15.1 *N* = 90 AD Age: 74.2 ± 8.2 *N* = 90 MCI Age: 70.5 ± 8.0 *N* = 33 FTD Age: 68.4 ± 7.0 *N* = 34 VaD Age: 73.2 ± 7.9	Rasch model	Single latent dimension reflecting a general cognitive functioning factor.	Infit/Outfit: most items had values within the optimal ranges (0.5–1.5). Only the first substraction item showed a moderate misfit (Outfit > 1.5). PSR = 0.85; PSI = 2.39.
Arcuri et al. (2015)	N = 74 with cancer diagnoses and MCI Age: 74.3% < 65 years	Partial Credit Model (PCM)	Single latent dimension reflecting a general cognitive functioning factor.	Infit/Outfit: most items had values within the optimal ranges (the item infit residual values ranged from 0.85 to 1.22 and outfit residuals from 0.41 to 1.43). The items City, Place, and Year showed misfit with the model because the difficulty levels were too easy for the sample.
de Paula Brandão et al. (2025)	N = 484 PD Age: 59.9 ± 11.1	Graded Response Model (GRM)	Single latent dimension reflecting a general cognitive functioning factor.	Attention had the highest discrimination (*aj* = 1.985), followed by Naming (*aj* = 1.740). Memory showed the lowest discrimination power (*aj* = 1.265).
Tsai et al. (2012)	*N* = 207 71 MCI Age: 79.2 ± 6.8, 98 AD Age: 79.6 ± 6.4, 38 HG Age: 77.7 ± 6.0	N.D.	Five domains: (1) Memory, (2) Visuospatial Skills, (3)Executive Functions, (4) Language, (5) Orientation.	The Memory, Visuo-spatial and Language domains were the steepest of the slopes and provided high discrimination power. The Orientation domain showed the lowest difficulty power (−2 to 0 SD).
Aiello et al. (2022)	*N* = 579 HG from Northern Italy Age: 63.4 ± 15.0	Two-parametr logistic (2 PL)	Single latent dimension reflecting a general cognitive functioning factor.	Trial Making Test and Delayed Recall had higher discrimination (*aj* = 1.527 and 1.962, respectively). The Orientation items (Month *bj* = −5.985) were the easiest.
Luo et al. (2020)	1873 older adults Age: 79.3 ± 8.0	Graded Response Model (GRM)	Three factors: (1) Visuospatial/executive, (2) Memory, (3) Language/Attention	Item Characteristic and Information Curves Orientation showed that Serial subtraction, and Naming had the highest curves, with high discriminatory power. Clock Number and Verbal fluency had very low curves and indicate poor discriminatory power. DIF for level education showed as Cube, Clock Number, and Clock Hand were more informative in the group without formal education.

HG, Healthy Group; AD, Alzheimer’s Disease; PD, Parkinson’s Disease; MCI, Mild Cognitive Impairment; DIF, Differential Item Functioning; GRM, Graded Response Model. 2PL, Two Parameters; PSR, Person Separation Reliability; PSI, Person Separation Index; SD, Standard Deviation.

Freitas et al. (2014) applied the Rasch model to a Portuguese sample (*N* = 897), estimating the item difficulty parameter (*bj*). The parameter *bj* indicates the latent ability level *θ* required to have a 50% probability of responding correctly (Balsamo et al., 2018a; Balsamo et al., 2018b). The Language Fluency item was the most difficult (*bj* = 2.32), indicating that only participants with a cognitive ability level significantly above average have a 50% probability of answering correctly. Conversely, the Orientation (city) item was the easiest (*bj =* −4.98), meaning that even participants with low cognitive ability have high probability of correct performance. Differential Item Functioning (DIF) analyses exhibited item stability across age, gender, education, and clinical group, supporting the generalizability of the MoCa. Infit statistics ranged between 0.75 and 1.29 and outfit statistics ranged from 0.44 to 1.68. Values within the 0.5–1.5 interval indicate that the items function consistently with Rasch model expectations (Linacre, 2011). The MoCA also showed high reliability (Person Separation Reliability = 0.85 and Person Separation Index = 2.39), indicating good identification across different ability levels.

Aiello et al. (2022) employed the GRM to a healthy Northern Italian sample (*N* = 579), estimating both item difficulty (*bj*) and discrimination (*aj*) parameters. The *aj* parameter indicates the ability of an item to discriminate among individuals with different levels of the latent trait θ (Balsamo et al., 2018a; Balsamo et al., 2018b). Values ≥ 1.35 are considered high discrimination (Baker, 2001). The most discriminative items were the Trial Making Test (Visual/Executive) (*aj* = 1.527), the Language Repetition of first sentence (*aj* = 1.691), and City (*aj* = 1.732), Place and Month in the Orientation domain showed low discrimination (*aj* = 0.034 and *aj* = 0.772, respectively). The least difficult items were the Clock Drawing Test –Contour (*bj* = −5.416), Attention Letter Detection Task (*bj* = −11.22), Month, and City (*bj* = −5.984 and *bj* = −4.157, respectively).

Arcuri et al. (2015) employed the PCM in a clinical sample of cancer patients with MCI. Item difficulty values ranged from −4.08 (Year, Place, and City in the Orientation domain) to 2.49 (Drawing of the cube). Infit and Outfit statistics fell within the optimal ranges, with the item infit residual values ranged from 0.85 to 1.22 and outfit residuals from 0.41 to 1.43. The Person Separation Reliability was 0.52, indicating low reliability, and the Person Separation Index of 1.04 suggested a poor ability to differentiate among different ability levels.

de Paula Brandão et al. (2025) used a GRM model to a sample of patients with Parkinson’s disease. The most discriminative domains were Attention (*aj* = 1.985) and Naming (*aj* = 1.740), while Memory showed the lowest discrimination (*aj* = 1.265) but the highest difficulty (*bj* = −0.860 to 2.402). DIF analyses showed that Memory Recall and Executive/Visuospatial domains were affected by participants’ educational level.

Luo et al. (2020) also employed the GRM model in a sample of Chinese older adults with low education level. Item Information Fuction (IIFs) showed that the Trial Making Test, Clock Numbers, and Clock Hands had the highest discriminative power, whereas Cube item was the most difficult. DIF analyses further indicated a significant effect of educational level on performance across several items.

Tsai et al. (2012) applied IRT to a Taiwanese outpatient sample. The Frontal/Executive and Language domains showed the strongest discrimination across most of the the ability spectrum, with discrimination parameters generally exceeding 1.50. The Memory domain had moderate discrimination (value around 1.20), with difficulty parameter clustered between −0.5 and 1.5. The Orientation domain had the lowest difficulty values (*bj* = −3.0 to −1.5).

Previous studies have shown that some domains (e.g., Orientation) have lower discriminative power (Arcuri et al., 2015; de Paula Brandão et al., 2025). Several authors have further pointed out that certain Orientation and Language items are more informative for advanced stages of pathology, while Memory appears to be less informative across the ability continuum (Tan et al., 2021; Tsai et al., 2012).

### Short forms of MoCA

Analyses of MoCA short forms developed using IRT consistently support the unidimensional structure of the test (Roalf et al., 2016; Bezdicek et al., 2020; Tan et al., 2021) (see Table 3). Roalf et al. (2016) identified an eight-item short form comprising Clock Draw, Trail making, Attention Digit Backwards, Attention Substraction, Language Fluency, Abstraction, Delayed Recall, Naming (rhino), and Orientation (place) items. This structure partially overlapped with the Czech version (Bezdicek et al., 2020), which differed specifically Orientation (place) and Naming (rhino). Tan et al. (2021) derived a six-item short form including Trail Making, Cube Copy, Attention Digit Span, Language Fluency, Clock Draw, and Delayed Recall. Across all studies, the selected items demonstrated adequate capacity to distinguish among different cognitive levels.

**Table 3:** Short versions of the MoCA.

Study	P-population	I-intervention	C-comparison	O-outcome
Roalf et al. (2016)	*N* = 1850 (MCI, AD, PD) Age: 70 ± 10.15	Graded Response Model (GRM)	One-factor second order model: (1) Visuospatial/ Executive, (2) Naming, (3) Attention, (4) Language, (5) Abstraction, (6) Recall Memory, (7) Orientation. Higher-order global cognition factor named “Cognition.”	Item included: Clock Draw, Trail Making, Attention Serial Subtraction, Language Fluency, Abstraction, Delayed Recall, Naming (rhino), Orientation (place). Item Characteristic and Information Curves Orientation showed that Serial subtraction, Clock Draw, Trail Making, and Delayed Recall had the highest curves, with high discriminatory power. Serial subtraction, Clock Draw, and Delayed Recall were the most difficult (0 to 3 SD).
Tan et al. (2021)	*N* = 4.007 HG *N* = 2.205 MCI *N* = 769 Dementia Age: from 80 to 90 Age: Mean ± SD unavailable.	Graded Response Model (GRM)	One-factor second order model: (1) Visuospatial/ Executive, (2) Naming, (2) Attention, (3) Language, (4) Abstraction, (5) Recall Memory, (6) Orientation. Higher-order global cognition factor named “Cognition.”	Item included: Trail Making, Cube Copy, Attention Digit Span, Language Fluency, Clock Draw, Delayed Recall. Item Characteristic and Information Curves Orientation showed that Serial subtraction, Clock Draw and Language Fluency had the highest curves, with high discriminatory power. Language Fluency was the most difficult (0 to 3 SD).
Hemrungrojn et al. (2024)	N = 60 HG Age: 67.9 ± 6.4 N = 61 MCI Age: 72.1 ± 7.0 *N* = 60 AD Age: 76.8 ± 8.0	Partial Least Squares (PLS)	One-factor second order model: (1) Visuospatial/ Executive, (2) Naming, (3) Attention, (4) Language, (5) Abstraction, (6) Recall Memory, (7) Orientation. Higher-order global cognition factor named “Cognition.”	Item included: Clock Time, Subtract 7, Fluency, Month, Year. All items loaded significantly on the latent factor, with loadings ranging from 0.571 and 0.763 (*p* < 0.01), supporting the internal consistency of the instrument (Cronbach’s Alpha = 0.832).
Bezdicek et al. (2020)	Total sample Czech Sample *N* = 699 HG Age: 71.27 ± 14.24 American sample *N* = 175 HG Age: 71.96 ± 9.34 *N* = 102 PD Age: 60.47 ± 8.54	Graded Response Model (GRM)	One-factor second order model: (1) Visuospatial/ Executive, (2) Naming, (3) Attention, (4) Language, (5) Abstraction, (6) Recall Memory, (7) Orientation. Higher-order global cognition factor named “Cognition.”	Item included: Clock Draw, Trail Making, Attention Serial Subtraction, Language Fluency, Abstraction, Delayed Recall, Digit Span Backwards, Language Repetition. Orientation Day and Attention Subtraction had higher discrimination (*aj* = 0.88 and 0.78, respectively). The Clock Draw (*bj* = −3.56) was the easiest.

HG, Healthy Group; AD, Alzheimer’s Disease; PD, Parkinson’s Disease; MCI, Mild Cognitive Impairment; DIF, Differential Item Functioning; GRM, Graded Response Model; SD, Standard Deviation; PLS, Partial Least Square.

Finally, Hemrungrojn et al. (2024) identified 5-item MoCA brief in a sample of 181 Thai older adults: Clock Time, Subtract 7, Fluency, Month, and Year.

## Discussion and conclusions

The present study examined the factorial structure of the MoCA using both CTT and IRT approaches. Overall, the results confirmed that the MoCA captures a broad set of cognitive domains. However, previous research has reported notable variability in its dimensional structure across studies and populations. Within the CTT framework, findings regarding the factorial structure have been notably inconsistent. For instance, Duro et al. (2010) proposed a two-factor model, whereas several studies have supported a second-order model in which first-order cognitive domains loaded onto a higher-order global cognition factor (Coen et al., 2016; Freitas et al., 2012; Karim and Venkatachalam, 2022). Additional evidence for a hierarchical model was provided by Sala et al. (2020), who identified seven cognitive domains contributing to a general cognitive factor. The IRT approach has identified specific items and domains of the MoCA with variability in discriminative and difficulty parameters. Performance in the Executive and Visuospatial domains consistently demonstrated strong discriminative properties across studies. Tasks such as the Trail Making Test and the Clock Drawing were among the most discriminative indicators of cognitive ability, displaying high discrimination parameters and the Cube was identified as the most difficult task (Luo et al., 2020). Aiello et al. (2022) further reported that the items exhibiting the least difficulty were the Clock Drawing Test –Contour. The Memory domain exhibited higher item difficulty, suggesting that correct responses require relatively elevated levels of cognitive ability. As noted by de Paula Brandão et al. (2025) and Tsai et al. (2012), memory items have been shown to be particularly informative for differentiating normal aging from MCI. This finding aligns with the well-established evidence that episodic memory—as assessed by the Memory Recall task—is particularly sensitive to decline during the prodromal stages of Alzheimer’s disease (Lee, 2023). On the other hand, language items demonstrated more variable discriminative power across samples. Repetition and verbal fluency tasks emerged as some of the most discriminative items of cognitive functioning (Aiello et al., 2022; Tsai et al., 2012). In contrast, naming and abstraction items demonstrated less consistent performance, particularly in lower-educated groups. These findings showed that the Language domain is indicative not only of cognitive functioning but also of sociolinguistic factors. This underscores the need for population-specific norms and potential adaptations for non-native speakers. The Attention domain generally demonstrated moderate to high discrimination across the ability continuum, whereas items belonging to the Orientation domain (City, Place, Month, Day, Year, Week) consistently exhibited low difficulty and limited discriminative power.

The findings of this study demonstrate that Executive and Visuospatial tasks, such as the Trial Making Test and the Clock Drawing, along with certain Language items (e.g., Naming), exhibited a greater capacity to differentiate across levels of the latent trait *θ*. In contrast, Language Fluency and Memory Recall tasks present the highest levels of difficulty. Moreover, the Orientation domain showed both low difficulty and low discrimination, sugesting a ceiling effect.

Substantial variation was observed across the reviewed studies in how authors addressed the heterogeneity of MoCA response format. Most authors (Bezdicek et al., 2020; de Paula Brandão et al., 2025; Freitas et al., 2014; Luo et al., 2020; Roalf et al., 2016; Tan et al., 2021; Tsai et al., 2012) retained the original scoring method, although that such a method might diminish the uniformity of items limiting the comparability of the derived parameters. In contrast, Aiello et al. (2022) dichotomized all items prior to applying the GRM, resulting in a uniform metric that simplifies cross-item comparison and model estimation. Nonetheless, for purposes of early detection and fine-grained profiling of cognitive abilities, retaining polytomous response categories is generally preferable as it preserves informational richness. Standardized scoring strategies ensure the comparability of item parameters and the aggregation of results to produce generalizable norms based on latent trait levels. As highlighted by Kline (2005) and Embretson and Reise (2013), psychometric validity depends on the use of a common metric and a coherent dimensional structure. When these conditions are not met, both measurement and construct validity may be undermined. Additionally, without a uniform and theoretically justified metric, parameter interpretation becomes less robust, potentially diminishing diagnostic accuracy.

### Clinical implications

From a clinician’s perspective, identifying the MoCA items and dimensions that exhibit the highest discrimination *aj* and difficulty *bj* parameters provides evidence-based guidance to enhance the effectiveness of cognitive screening procedures. Items with high discrimination are particularly sensitive to minimal differences in cognitive ability and are therefore crucial for the early detection of MCI. Conversely, high-difficulty items are more likely to be failed by participants with more pronounced deficits, making them especially informative in case of moderate to severe stages of cognitive impairment.

Conversely, items with low discriminative and difficulty parameters provide reduced utility for the early detection of cognitive decline. This is due to the fact that even participants with mild impairment typically tend to respond correctly to these items, increasing the likelihood of false negatives in the early stages of pathology (Embretson and Reise, 2013). Clinicians should interpret each domain as reflecting specific cognitive processes that may guide differential diagnosis.

Items with high discrimination are particularly sensitive to small differences in cognitive ability, and their relevance for early diagnosis is further einforced when cross-validation analyses exhibits that these items have high discriminative power across independent samples (Wen et al., 2025).

The Executive, Visuospatial, and Attentional domains demonstrated the highest discriminative power, confirming their sensitivity to early cognitive decline. Language and Memory tasks were found to be more difficult, reflecting their reliance on higher-order cognitive and educational background. In contrast, Orientation items showed both low discrimination and difficulty, suggesting limited diagnostic utility except in more advanced stages of impairment. From a clinical perspective, it is essential to interpret each domain as reflecting specific cognitive processes that can inform differential diagnosis and guide early intervention strategies. For instance, low performance in Executive or Visuospatial tasks may signal early-fronto-subcortical or attentional dysfunction, whereas difficulties in Deylayed Recall or Language Repetition may indicate imapairment in the temporal or semantic networks (Ma et al., 2023). The cognitive deficits observed in individuals with MCI can be well-established predictors of progression to Alzheimer’s disease (Mieling et al., 2025; Spalletta et al., 2014). A recent meta-analysis by Salemme et al. (2025), including 89 studies, reported conversion rates of 41.5% in clinical cohorts and 27.0% in population-based studies. Such evidence is essential for informing early, targeted interventions aimed at modifying risk and protective factors. In conclusion, the present study contributes to the field by integrating CTT and IRT evidence to clarify how individual MoCA items and domains function psychometrically and clinically. The findings highlight domain-specific patterns of difficulty and discrimination, emphasize the importance of sociodemographic influences, and underscore the need for methodological rigor in scoring and interpretation. Together, these insights support more accurate cognitive profiling and more effective clinical screening for early cognitive impairment.
